# Understanding rapid oral health deterioration and its associated factors among older adults: A scoping review

**DOI:** 10.12688/f1000research.149120.1

**Published:** 2024-04-17

**Authors:** Radhika Ranjith, Ramya Shenoy, Parul Dasson Bajaj, Ashwini Rao, Mithun Pai, Praveen Jodalli, Avinash BR, Harsh Priya, Navya Shinaj, Violet D'Souza

**Affiliations:** 1 Department of Public Health Dentistry, Manipal College of Dental Sciences Mangalore, Manipal Academy of Higher Education, Manipal, Karnataka, 576104, India; 2 Department of Public Health Dentistry, CDER, AIIMS, New Delhi, India; 3 Department of Dental Clinical Sciences, Faculty of Dentistry, University of Dalhousie, Halifax, NS B3H 4R2, Canada

**Keywords:** Health and wellbeing, clinical deterioration, Oral health deterioration, Aged, Older adults

## Abstract

**Background:**

Understanding the pivotal interplay between systemic and oral health is paramount to ensuring holistic care, particularly among the aging demographic. Therefore, this review article aims to explore the emerging concept of Rapid Oral Health Deterioration (ROHD) by reviewing the current knowledge base among older adults and identifying knowledge gaps in this area of research.

**Methods:**

This scoping review was conducted in line with Arksey and O’Malley’s framework between December 2023 and March 2024 and reported while adhering to the PRISMA-ScR guidelines. A systematic database search was performed across three databases i.e. PubMed, Scopus, and EMBASE to collate the existing literature published in English between January 2013 and February 2024 addressing ROHD among older adults. After data charting, a critical appraisal of the selected studies was followed by qualitative thematic analysis.

**Results:**

Among the 12 papers in this scoping review, 10 were cross-sectional studies, with one each of retrospective cohort and case-control studies. The qualitative thematic analysis of the selected articles resulted in the emergence of four main themes: risk factors for ROHD, attributes related to ROHD, challenges encountered in the management of ROHD, and management approaches for ROHD among older adults.

**Conclusions:**

This scoping review provides an overview of the rapid deterioration of oral health among older adults. Age-related dental disease harms the quality of life and overall health. To avoid dental disorders and to maintain and improve oral health in older adults, an integrated and multidisciplinary approach is essential. If ROHD is not treated, it may lead to poor health, a lower quality of life, and in severe cases, systemic infections that increase hospitalizations and possibly cause death.

## Introduction

The aging demographic is an enduring worldwide phenomenon, with projections indicating that by 2050, one out of every six individuals globally will be aged 65 or above, compared to one in ten in 2021. ^1^ As individuals age, they become more vulnerable to a variety of systemic illnesses, with the older adults exhibiting a significantly higher prevalence of chronic and non-communicable diseases compared to the younger demographic. ^2^ It is often suggested that the condition of the oral cavity reflects a person’s general health. ^3^^,^^4^ Oral diseases, particularly periodontal disease, have been linked to various non-communicable conditions like diabetes, cardiovascular diseases, cancer, cognitive decline, among others. ^5^^,^^6^ Therefore, recognizing the vital relationship between overall health and oral health is essential for safeguarding an individual’s overall well-being, as general health status significantly influences oral health outcomes. ^3^

Although the complete loss of teeth may be declining, gradual tooth loss remains a significant indicator of poor oral health in older adults with dental caries continuing to be the most prevalent oral condition among older individuals. ^5^^,^^6^ Hence, early detection and treatment of oral health issues in older adult patients can mitigate oral health complications, particularly in those with chronic illnesses, and contribute to enhancing overall Oral Health-Related Quality of Life. ^7^

The care needs of older individuals are diverse and intricate, which is highlighted by the emergence of medical and dental specializations that prioritize patient-centered care. Programs in geriatric dentistry specifically aim to understand the interplay between oral health conditions, socioeconomic factors, and systemic health issues in this demographic. ^8^ Common chronic systemic disorders diagnosed in older individuals include arthritis, hypertension, diabetes, depression, neurodegenerative conditions, dementia, and stroke. Recognizing potential interactions between oral health factors and these systemic health events is crucial for addressing the poor oral health encountered in this age-group. This serves as the foundational premise of the systematic framework outlined in the Rapid Oral Health Deterioration (ROHD) concept, aiding dental professionals in assessing the probability of progressive oral health deterioration among older adult patients. ^6^^,^^9^

Understanding the potential the concept of ROHD holds in alleviating the oral disease burden among older adults, the aim of this scoping review was to explore the existing literature on ROHD in older adults, an area that has received relatively limited attention in research. The objective was to compile and analyze the literature while also pinpointing and comprehending the existing knowledge gaps in this realm of research.

## Methods

The time frame for this scoping review ranged between December 2023 and March 2024 following the framework outlined by Arksey and O’Malley, ^10^ to address the research question “What is known about rapid oral health deterioration among older adults in the existing literature?” A review protocol was not registered for this scoping review, and the findings are presented in accordance with the PRISMA-ScR checklist. ^11^

### Eligibility criteria

This scoping review included articles focusing on one or more facets of oral health deterioration in older adults, published in English between January 2013 and February 2024 reflecting on the heightened awareness around oral health needs of the geriatric population. Review articles, letters to editors, and other papers with missing full-text formats, such as conference proceedings, were excluded from this scoping review.

### Information sources and search strategy

A systematic search was conducted across three databases i.e. PubMed, Scopus, EMBASE, where the last search was performed in February 2024. The search strategy involved using key terms such as ‘older adults’, ‘older adults’, ‘aged’, ‘clinical deterioration’ and ‘rapid oral health deterioration’, along with Boolean operators ‘OR’ and ‘AND’. The detailed search strategy across the databases has been provided in Table 1.

**Table 1.** The detailed search strategy across the databases.

Data base	Fields chosen	Date accessed	Search strategy	Results popping out	Final
PUBMED	All fields #1	28 ^th^ Jan 2024	Search: ((((((((aged) OR (elderly)) OR (older adults)) AND (clinical deterioration)) OR (oral health)) OR (root caries)) OR (tooth loss)) OR (rapid oral health deterioration)) AND (india) (((“aged”[MeSH Terms] OR “aged”[All Fields] OR (“aged”[MeSH Terms] OR “aged”[All Fields] OR “elderly”[All Fields] OR “elderlies”[All Fields] OR “elderly s”[All Fields] OR “elderlys”[All Fields]) OR (“aged”[MeSH Terms] OR “aged”[All Fields] OR (“older”[All Fields] AND “adults”[All Fields]) OR “older adults”[All Fields])) AND (“clinical deterioration”[MeSH Terms] OR (“clinical”[All Fields] AND “deterioration”[All Fields]) OR “clinical deterioration”[All Fields])) OR (“oral health”[MeSH Terms] OR (“oral”[All Fields] AND “health”[All Fields]) OR “oral health”[All Fields]) OR (“root caries”[MeSH Terms] OR (“root”[All Fields] AND “caries”[All Fields]) OR “root caries”[All Fields]) OR (“tooth loss”[MeSH Terms] OR (“tooth”[All Fields] AND “loss”[All Fields]) OR “tooth loss”[All Fields]) OR ((“rapid”[All Fields] OR “rapidities”[All Fields] OR “rapidity”[All Fields] OR “rapidness”[All Fields]) AND (“oral health”[MeSH Terms] OR (“oral”[All Fields] AND “health”[All Fields]) OR “oral health”[All Fields]) AND (“deteriorate”[All Fields] OR “deteriorated”[All Fields] OR “deteriorates”[All Fields] OR “deteriorating”[All Fields] OR “deterioration”[All Fields] OR “deteriorations”[All Fields] OR “deteriorative”[All Fields])) OR (“oral hygiene”[MeSH Terms] OR (“oral”[All Fields] AND “hygiene”[All Fields]) OR “oral hygiene”[All Fields])) AND (“india”[MeSH Terms] OR “india”[All Fields] OR “india s”[All Fields] OR “indias”[All Fields])	13353	10
	#2	28 ^th^ Jan 2024	Search: (((((((((((((aged) OR (elderly)) OR (older adults))) AND (clinical deterioration)) OR (oral health)) OR (root caries)) OR (tooth loss)) OR (rapid oral health deterioration)) OR (oral hygiene)) AND (india) ((((“aged”[MeSH Terms] OR “aged”[All Fields] OR (“aged”[MeSH Terms] OR “aged”[All Fields] OR “elderly”[All Fields] OR “elderlies”[All Fields] OR “elderly s”[All Fields] OR “elderlys”[All Fields]) OR (“aged”[MeSH Terms] OR “aged”[All Fields] OR (“older”[All Fields] AND “adults”[All Fields]) OR “older adults”[All Fields])) AND (“clinical deterioration”[MeSH Terms] OR (“clinical”[All Fields] AND “deterioration”[All Fields]) OR “clinical deterioration”[All Fields])) OR (“oral health”[MeSH Terms] OR (“oral”[All Fields] AND “health”[All Fields]) OR “oral health”[All Fields]) OR (“root caries”[MeSH Terms] OR (“root”[All Fields] AND “caries”[All Fields]) OR “root caries”[All Fields]) OR (“tooth loss”[MeSH Terms] OR (“tooth”[All Fields] AND “loss”[All Fields]) OR “tooth loss”[All Fields]) OR ((“rapid”[All Fields] OR “rapidities”[All Fields] OR “rapidity”[All Fields] OR “rapidness”[All Fields]) AND (“oral health”[MeSH Terms] OR (“oral”[All Fields] AND “health”[All Fields]) OR “oral health”[All Fields]) AND (“deteriorate”[All Fields] OR “deteriorated”[All Fields] OR “deteriorates”[All Fields] OR “deteriorating”[All Fields] OR “deterioration”[All Fields] OR “deteriorations”[All Fields] OR “deteriorative”[All Fields])) OR (“oral hygiene”[MeSH Terms] OR (“oral”[All Fields] AND “hygiene”[All Fields]) OR “oral hygiene”[All Fields])) AND (“india”[MeSH Terms] OR “india”[All Fields] OR “india s”[All Fields] OR “indias”[All Fields])) AND (2014:2024[pdat])	0	0
	#3	28 ^th^ Jan 2024	Search: **(rapid oral health deterioration) AND (older adults)** (“rapid”[All Fields] OR “rapidities”[All Fields] OR “rapidity”[All Fields] OR “rapidness”[All Fields]) AND (“oral health”[MeSH Terms] OR (“oral”[All Fields] AND “health”[All Fields]) OR “oral health”[All Fields]) AND (“deteriorate”[All Fields] OR “deteriorated”[All Fields] OR “deteriorates”[All Fields] OR “deteriorating”[All Fields] OR “deterioration”[All Fields] OR “deteriorations”[All Fields] OR “deteriorative”[All Fields]) AND (“aged”[MeSH Terms] OR “aged”[All Fields] OR (“older”[All Fields] AND “adults”[All Fields]) OR “older adults”[All Fields]) **Translations rapid:** “rapid”[All Fields] OR “rapidities”[All Fields] OR “rapidity”[All Fields] OR “rapidness”[All Fields] **oral health:** “oral health”[MeSH Terms] OR (“oral”[All Fields] AND “health”[All Fields]) OR “oral health”[All Fields] **deterioration:** “deteriorate”[All Fields] OR “deteriorated”[All Fields] OR “deteriorates”[All Fields] OR “deteriorating”[All Fields] OR “deterioration”[All Fields] OR “deteriorations”[All Fields] OR “deteriorative”[All Fields] **older adults:** “aged”[MeSH Terms] OR “aged”[All Fields] OR (“older”[All Fields] AND “adults”[All Fields]) OR “older adults”[All Fields]	35	9
	#4	28 ^th^ Jan 2024	Search: **rapid oral health deterioration** (“rapid”[All Fields] OR “rapidities”[All Fields] OR “rapidity”[All Fields] OR “rapidness”[All Fields]) AND (“oral health”[MeSH Terms] OR (“oral”[All Fields] AND "health”[All Fields]) OR “oral health”[All Fields]) AND (“deteriorate”[All Fields] OR “deteriorated”[All Fields] OR “deteriorates”[All Fields] OR “deteriorating”[All Fields] OR “deterioration”[All Fields] OR “deteriorations”[All Fields] OR “deteriorative”[All Fields]) **Translations** **rapid:** “rapid”[All Fields] OR “rapidities”[All Fields] OR “rapidity”[All Fields] OR “rapidness”[All Fields] **oral health:** “oral health”[MeSH Terms] OR (“oral”[All Fields] AND “health”[All Fields]) OR “oral health”[All Fields] **deterioration:** “deteriorate”[All Fields] OR “deteriorated”[All Fields] OR “deteriorates”[All Fields] OR “deteriorating”[All Fields] OR “deterioration”[All Fields] OR “deteriorations”[All Fields] OR “deteriorative”[All Fields]	82	10
	#5	28 ^th^ Jan 2024	Search: **((older adults) AND (clinical deterioration)) AND (oral health)** (“aged”[MeSH Terms] OR “aged”[All Fields] OR (“older”[All Fields] AND “adults”[All Fields]) OR “older adults”[All Fields]) AND (“clinical deterioration”[MeSH Terms] OR (“clinical”[All Fields] AND “deterioration”[All Fields]) OR “clinical deterioration”[All Fields]) AND (“oral health”[MeSH Terms] OR (“oral”[All Fields] AND “health”[All Fields]) OR “oral health”[All Fields]) **Translations** **older adults:** “aged”[MeSH Terms] OR “aged”[All Fields] OR (“older”[All Fields] AND “adults”[All Fields]) OR “older adults”[All Fields] **clinical deterioration:** “clinical deterioration”[MeSH Terms] OR (“clinical”[All Fields] AND “deterioration”[All Fields]) OR “clinical deterioration”[All Fields] **oral health:** “oral health”[MeSH Terms] OR (“oral”[All Fields] AND “health”[All Fields]) OR “oral health”[All Fields]	255	12
SCOPUS	All fields #1	28 ^th^ Jan 2024	aged AND elderly AND “Geriatric dentistry" AND “older adults" AND “oral health deterioration" AND “Rapid oral health deterioration" AND “Tooth loss" AND “Root caries" AND “clinical deterioration"	0	0
	#2		"Geriatric dentistry" AND “Rapid oral health deterioration" AND “Tooth loss" AND “Root caries" AND “clinical deterioration"	0	0
	#3		"Geriatric dentistry" AND “Rapid oral health deterioration"	6	5
	#4		"Geriatric dentistry" AND “oral health deterioration" AND “Tooth loss" AND “Root caries"	9	6
EMBASE	All fields #1	14 ^th^ Feb 2024	('older adults'/exp OR 'older adults' OR (older AND ('adults'/exp OR adults))) AND ('aged'/exp OR aged) AND ('elderly'/exp OR elderly) AND ('geriatric dentistry'/exp OR 'geriatric dentistry' OR (('geriatric'/exp OR geriatric) AND ('dentistry'/exp OR dentistry)))	1603	14
	#2		('clinical deterioration'/exp OR 'clinical deterioration' OR (('clinical'/exp OR clinical) AND ('deterioration'/exp OR deterioration))) AND ('oral health deterioration' OR (oral AND ('health'/exp OR health) AND ('deterioration'/exp OR deterioration)))	888	7
	#3		('clinical deterioration'/exp OR 'clinical deterioration' OR (('clinical'/exp OR clinical) AND ('deterioration'/exp OR deterioration))) AND ('tooth loss'/exp OR 'tooth loss' OR (('tooth'/exp OR tooth) AND ('loss'/exp OR loss))) AND ('oral health deterioration' OR (oral AND ('health'/exp OR health) AND ('deterioration'/exp OR deterioration)))	152	13

### Selection process

The initial database search was conducted by one author (RR) using the search strategy, followed by the removal of duplicates. Title and abstract screening were independently performed by two investigators (RR and RS), with each article categorized as “Yes”, “No”, or “Maybe”. Subsequently, the articles selected during title and abstract screening underwent full-text review by the two investigators independently. Disagreements were resolved through discussion at each stage, leading to consensus. The articles which fulfilled the eligibility criteria were included in this scoping review for data extraction and critical appraisal.

### Data charting process

One author (RR) charted the data from the selected full-text articles using a predetermined format, which was followed by random verification by another author (RS). The predetermined format encompassed the following headings: title of the study and the authors, year of publication, purpose of theoretical and conceptual orientation, research questions and/or hypothesis, sample description, methods and analysis, key measures, main findings or results, author(s) stated limitations of the study, additional limitations of the study, strength and importance of the study.

### Critical appraisal of articles

The Crowe Critical Appraisal Tool (CCAT) ^12^ was employed to assess the quality of the available evidence and accurately identify the research gaps in the literature. CCAT enables the critical assessment of various research methodologies and evaluates each article in eight categories i.e. preliminaries, introduction, design, sampling, data collection, ethical issues, results and discussion. Each category is scored between from 0-5 and with a possibility of the maximum total score of 40. Based on the CCAT scores, articles with a score of 35 or more were labelled as high quality, scores from 25 to 34 were deemed medium quality while articles with scores less than 25 were considered as low quality. ^13^ The critical appraisal was executed by one author (RR), with a subset of articles randomly selected for reassessment by another author (RS) to ensure accuracy.

### Synthesis of results

The findings of this scoping review were synthesized through qualitative thematic analysis of each selected article. Two authors (RR and PDB) independently conducted the coding process for thematic analysis, where the appropriate segments of the selected articles pertaining to the research question on rapid oral health deterioration among older adults were identified and initially coded using Atlas.ti Mac ^14^ (Version 23.2.1). Subsequently, both authors deliberated on the initial codes to arrive at the final codes and at this juncture, a third author (RS) joined the discussion for further refinement of the codes and through comprehensive discussion, the main themes emerging from the selected articles addressing the research question were identified and organized into main themes and subthemes for enhanced clarity and comprehension.

## Results

**Figure 1.**
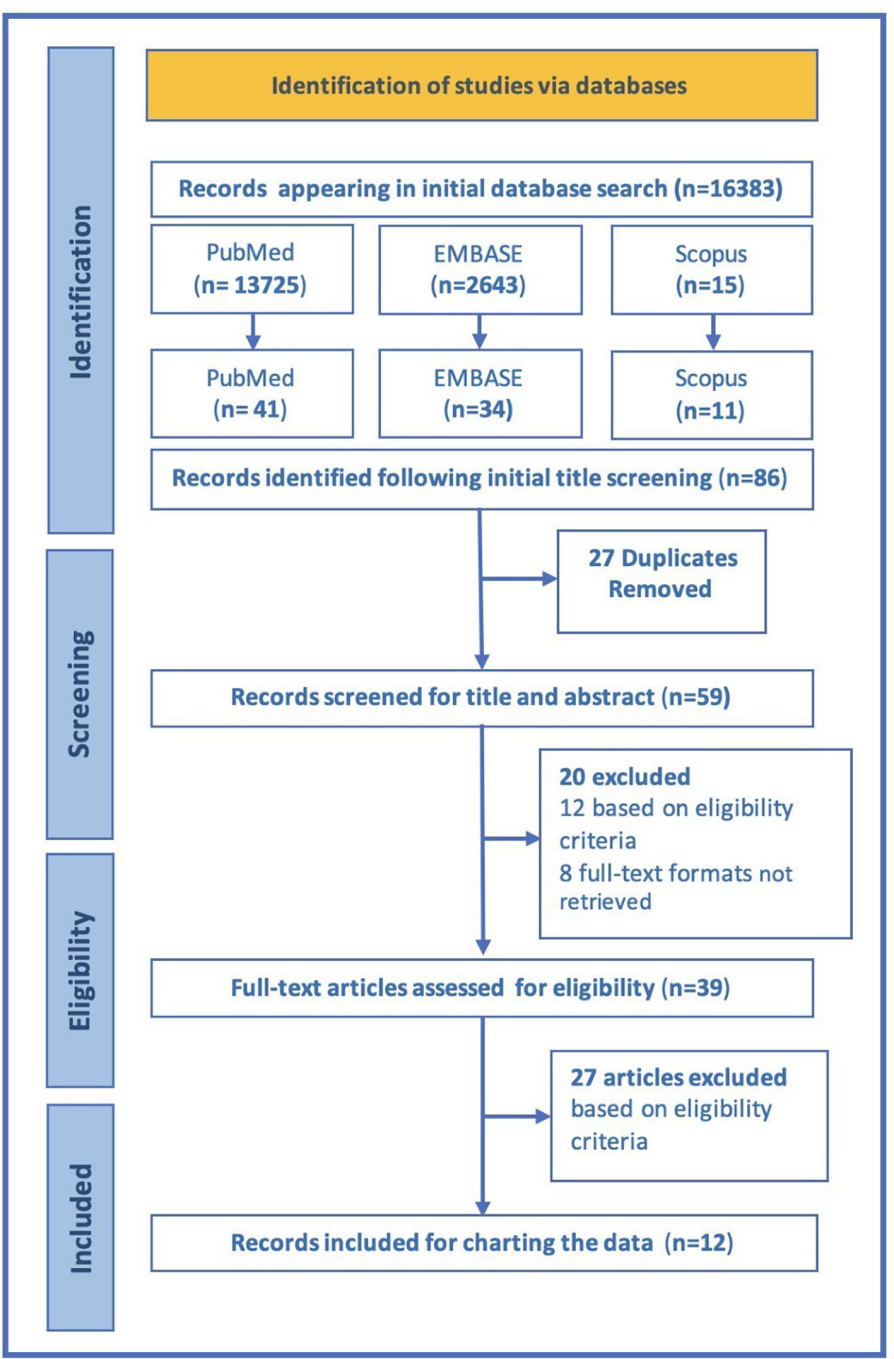
PRISMA flow diagram depicting the selection of articles.

A total of 86 articles were selected after the initial search based on title screening, of which 27 duplicates were eliminated. Subsequently, this was followed by a title and abstract screening of the remaining 59 articles, of which 39 were considered for a comprehensive full-text review. Based on the eligibility criteria, finally 12 articles were deemed suitable for inclusion in this scoping review. The selection process of the articles is illustrated in Figure 1 with a PRISMA flow diagram. ^15^

### Characteristics and quality of sources of evidence

Among the 12 papers included in this scoping review, ten were cross-sectional studies, with one each of retrospective cohort and case-control studies. These papers were mostly published within the last ten years, as per the eligibility criteria which included articles from 2013 onwards. Table 2 offers a comprehensive overview of the characteristics of the articles selected for this scoping review, along with the data extracted from these sources.

**Table 2.** Charted data from the included studies.

Title, Author(s) and Year of Publication	Purpose of theoretical and conceptual orientation	Research questions and/or Hypothesis	Sample Description	Methods and Analysis	Key Measures	Main Findings or Results	Author(s) stated limitations of the study	Additional limitations of the study	Strength and importance of the study
Dental caries in older adults in the last year of life Chen et al. 2013 ^16^	The study was conducted to examine the association between dental caries severity being in the last year of life in older adults.		The study consisted if 1216 older adults aged 65 years or above which included 168 individuals in the last years of life.	The study was conducted by measuring the patients socio-economic status Medical history, medications, functional status, oral health history including periodontal status, dentate status, caries assessment were done. The National Death Index was used for determination of end-of-life status.	The dental caries severity in the last year of life adjusting the mobility, oral care capacity and death was done by negative binomial regression.	It was found that end-of-life participants had only slightly elevated and non-statistically significant risk than those not in the last year. Without the impair of the oral function the caries severity decreased among end-of-life participants.	There was a lack of uniform criteria for caries assessment. The dental caries and retained roots were grouped into one variable to overcome this. There might be a misestimation of the caries severity in the study participants.		
Oral hygiene and associated factors among frail older assisted living residents Saarela et al-2013 ^17^	The study aims to determine the tooth brushing habits with health and nutritional status of older assisted residents.		1475 residents in Helsinki.	This study assessed tooth brushing habits, nutritional status, oral health and the use of dental services among 1447 residents in Helsinki metropolitan area of Finland. Subjective health of the residents were assessed and graded as 1-4 where 1 is healthy and 4 being very healthy. SPSS and NCSS were used.	χ2-test or Fischer exact test for categorical variables and the Mann–Whitney U-test for continuous variables. And the participants who clean the teeth and denture on a daily basis were compared to the subjects who don’t.	It was found that the residents who didn’t clean their teeth and/or theirs dentures were 17%.They were males and less educated and had a mean length of stay in assisted living longer than the subjects who cleaned their teeth and dentures regularly. People with dementia were found to clean their teeth less when compared to the others.	This study could not understand the causal relationship between oral hygiene and vascular diseases.		The study population was found to be large and it included all residents in assisted living in metropolitan region of Finland. It provides an important association between tooth brushing, demographic details, oral health, general health nutrition etc.
Relationship between oral health and nutritional status in the older adults: A pilot study in Lebanon El Hélou et al. 2014 ^18^	The aim of the study is to understand the relationship between oral health and nutritional status of a Lebanese population of non-institutionalized older adults patients newly admitted to hospital for acute medical conditions.		The survey was conducted on 115 persons aged 70 years and older.	During three consecutive months the subjects were admitted in various wards of Rafic Hariri University hospital in Lebanon. Socioeconomic, medical, dietary, anthropometric data was collected. Mini-Nutritional assessment assessed the Nutritional status, Geriatric Oral Health Assessment Index assessed the oral health. Following which the oral cavity was examined to know about the remaining teeth, presence of dentures and its status, and assess xerostomia.		6.1% had a prevalence of undernutrition and 37.4% with an additional risk of malnutrition. Nutritional deficiency was a risk factor in more than 50% of individuals in need of dental care. Once the socioeconomic factors, neurosensory disorders and chronic diseases were adjusted the risk of nutritional deficiency disappeared.	Since the sample size was comparatively small the association was not significant.		This pilot study is designed to establish a protocol that is appropriate for a national survey.
Aging and oral care: An observational study of characteristics and prevalence of oral diseases in an Italian cohort Lauritano et al-2019 ^19^	The purpose of this study was to evaluate the features and prevalence of oral pathology in an older adults dementia-affected group. It also aimed to investigate the relationship between each person's oral health and the different degrees of dementia.		The sample size was 39. 34 women and 5 men	The observational study consists of 2 groups of older adults patients suffering from dementia from 2 different residential care. Clinical dementia rating scale was used to grade the level of dementia in the patients. Different oral parameters were assessed in these patients (number of remaining teeth, oral mucosa, periodontal tissues, bone crests). Scores were assigned to each of them. Spearman’s Rho test was used	Number of remaining teeth Oral mucosa Periodontal tissue Bone crests Level of cooperation Oral examination The correlation index (r) was calculated taking into consideration the oral health values of patients with CDR between 2 and 5 (moderate – severe – very severe – terminal dementia).	20.58% of the patients had mucosal lesions/new mucosal formations. A marked clinically evident reabsorption of bone crest was found in almost all patients 88.23%. The prevalence of periodontal disease was 82.35%. 24.13% of the patients who underwent oral examination had totally edentulous maxilla retained roots or without prosthetic rehabilitations. They found a linear correlation between the degree of dementia and the oral health of the patient.	The limited sample size and inadequate balance between the males and the females is a limitation.		This study is the same method that is used to perform oral examination and the recording all the social, behavioral and clinical information about the patients.
Teaching rapid oral health deterioration risk assessment: A 5-year report. Craig & Johnsen, 2020 ^20^	The study was done to present a 5-year report on the outcomes of using a teaching tool that guides dental students on how to assess the risk of ROHD		188 Senior fourth year students	The data was assessed by the performance data obtained from 188 students from their ROHD risk assessment seminars which was a part of their GSNDP rotation. Students had a presentation for 20 minutes(10 minutes presentation and 10 minute discussion) on the last day of GSNDP rotation. It was assessed by a faculty member and an examiner.	Students were graded A-student Applied the step, an objective assessment, G-student not only applied but also Grasped the principle behind the step, thus incorporating a subjective assessment, M- student did not address/missed the step.	The evaluation of the students suggests that 75% of them had A or G for most questions. The students performance was improved in the 5-year period. 94.4% of the students considered the teaching tool very important for general dentist.	The study was limited to one American dental school. The teaching tool cannot be utilized if the curriculum is different.	Lack of faculties and availability to provide multiple assessments.	It prepares the future dental workforce for treating the older population and the tool helps in approaching dentists effectively in geriatric dentistry.
Association Between Oral Health and Frailty Among American Older Adults Hakeem et al. 2021 ^21^	The objective of the study is to assess the association between tooth loss, periodontal diseases and frailty among American older adults.		The study consisted of 2368 community dwelling older adults of 60 years or older.	The frailty of the subjects was measured by the 49 item Frailty index. The number of teeth and the periodontal disease were the oral health indicators. The association between the oral health indicators and frailty was assessed by negative binomial regression.	The statistical analysis was done using Stata version 16. Negative binomial regression was used to test the association between frailty index and oral health indicators.	38.7% of the participants were frail. The current and previous smokers low percentage of high education and lower poverty income was among the frail participants. Inadequate nutrition intake was significantly associated with periodontitis.	The study was unable to draw the casual relationship between oral health indicators and frailty.	The data on comorbidities was self reported so they might be subject to recall bias.	This phenotype model emphasizes on the importance of maintaining oral health and natural teeth in older adults.
Oral health status in older people with dementia: A case—control study Lopez-Jornet et al. 2021 ^22^	This case-control study was aims to assess the impact of dementia on the oral health.		The study consists of 152 patients. 69 with dementia and 83 control.	An oral exploration was carried out using the Global Deterioration Scale to classify the patients. Regression models were used to calculate and estimate the Odds ratio and the Confidence interval.		The gingival bleeding, plaque index and fewer natural teeth were found in patients with more severe disease than the control group. It was found that there was a deficient oral health in patients with dementia.	One of the limitation of the study was the patients and the control may be suffering from other disease than dementia that would affect their oral health.		
Oral Health-Related Quality of Life, Oral Conditions, and Risk of Malnutrition in Older German People in Need of Care—A Cross-Sectional Study Schmalz et al. 2021 ^23^	This cross-sectional study conducted among older adults Germans requiring care to understand the relationship between their oral health, nutritional condition, oral health related quality of life.	The hypothesis set was the risk of malnutrition will be correlated with the older adults's oral health-related quality of life as well as poor oral health.	From eight nursing facilities, the participants were chosen and was estimated to be about 500. Based on the inclusion and the exclusion criteria it was estimated to be 151 older people.	The oral health status including the DMFT, root caries, requirement for periodontal therapy, and prosthetic problems were recorded. Mini nutritional assessment was used to assess the nutritional status. The oral health impact profile was used to evaluate quality of life as it relates to oral health. It was summarized as a total sum score and also as oral function, psychosocial impact, pain and orofacial appearance. Linear logistic regression was done.		Most participants 60.3% were nursing residents and 47% were edentulous. 74.4% of dentate participants required periodontal treatment. 115 of the subjects had at least one denture. 70.9% of the older adults had suffered from malnutrition or already had a risk of malnutrition.	The study design was extensive. Absence of a comparison group was considered as a limitation of the study. Xerostomia, dysphagia, burning mouth etc which were of high relevance to oral health related quality of life was not addressed in the study.		The study can be generalized as the sample size seems appropriate. This study provides information importance dental care in older adults and the findings of this study are clinically relevant.
Prevalence and Clinical Correlation of Decayed, Missing, and Filled Teeth in Older adults Inpatients With Schizophrenia Yang et al. 2021 ^24^	The aim of the study is to assess the oral health status of patients with schizophrenia, the related factors and to provide scientific evidence for further exploration of corresponding control strategies.		425 inpatients older than 50 years with schizophrenia from 2 psychiatric hospitals.	The mean age was 58.49+/- 5.72 years. The demographic details of the patients were checked the caries, missing teeth, fillings were examined by two independent dentists. The cognitive tests performed were Mini-Mental State Examination and Global Deterioration Scale.		The patients in the two hospitals showed a significant difference in the mean DMFT score. High DMFT was associated with age and smoking, Females and those with higher education level showed a lower DMFT score.	Only one dimension of DMFT was evaluated in this study. Chewing, swallowing, speech which are other oral functional indexes were not included. The study was a multicentered cross- sectional study and the regional difference may change the dietary habits and cause a biased result.		
Oral health and nutritional assessment among older adults -An explorative study Devika et al.,2023 ^25^	The study aims to assess the use of OHI-S, Russel’s periodontal index (PII), DMFT index to evaluate the oral status as well as to know about the nutritional status of the population.		The study consisted 66 participants including 30 older adults from old-age home and 36 were community dwelling.	The oral status of the population was evaluated for the OHI-S, PII, DMFT index. The nutritional status was assessed using the Mini Nutritional Assessment.		The mean BMI of the population was found to be 25.92+/- 3.90. The score of 38 participants fell within the normal limits and only 4 participants were malnourished.	The inavailability of medical reports made it not possible to assess the medical conditions and the drug history of the participants.		This was one among the very few studies that was done to understand the correlation of nutritional status with oral health among older adults.
The effects of dental visits on the occurrence of acute hospitalization for systemic diseases among patients aged 75 years or older: A propensity score-matched study Mitsutake et al. 2023 ^26^	The study aims to understand the effect of dental visits on prevention od the acute hospitalization due to systemic conditions such as pneumonia, UTI, cerebrovascular diseases, Coronary heart disease in older adults ages 75 years or older in Japan.		Study sample were the patients aged 75 years or older who have received dental and medical care under MCSO between September 2016 to February 2017. 148032 was the final sample size.	Occurrence of Acute hospitalization due to pneumonia, UTI, and cerebrovascular disease or coronary heart disease was the primary outcome variable.	The propensity score matching was done to compare the outcomes of patients who visited and did not visit dental office.	Among the patients who visited the dental office the occurrence of acute hospitalization due to pneumonia, UTI, and cerebrovascular disease or coronary heart disease were lower than among those who did not visit the dental office.	The study defined the occurrence of acute hospitalization due to systemic diseases using medical procedures from insurance claims data commonly used to treat each systemic disease in acute hospital care.	Due to the absence of any studies on the occurrence of acute hospitalization among Japanese aged 75 years or older due to systemic diseases the could not discuss the validity of occurrence of systemic disease in this study.	This study was able to provide important implication of healthcare among the older adults.
Oral frailty among dentate and edentate older adults in long-term care Julkunen et al. 2024 ^27^	The objective of the study was to compare the oral frailty among the dentate and edentate older adults living in long term care facilities and the association between oral disease burden and edentulism on oral frailty.		The study comprised of 303 residents of LTCF in Helsinki Finland. 94 edentate and 209 dentate residents.	An oral examination was carried out and the participants were divided into three oral disease burden groups on the basis of asymptotic dental score. The groups were compared regarding the demographic details, oral and the medical findings. A multivariate logistic regression model was employed to examine the relationship between edentulousness and oral frailty.		Oral frailty was found to be less significant in participants with low oral disease burden than their edentate peers. The odds for oral frailty was found to be similar in edentate and dentate with high oral disease burden.	The main limitation of the study is that it is a cross-sectional study and there is no information regarding the residents before admission to LTCF. There were relatively few participants with low oral disease burden.		The data of the study comprises a large population of older adults in a long term care where the oral health status was comprehensively examined by dentist

CCAT was used to conduct a quality appraisal of the selected sources of evidence, and the detailed scores for each paper across all domains are presented in Table 3. According to the CCAT scores, eleven papers included in the review were deemed as medium quality, while one was labelled as low quality based on the CCAT scores.

**Table 3.** Crowe’s critical appraisal scores of the included studies.

Author and year of publication	Preliminaries	Introduction	Design	Sampling	Data collection	Ethical	Results	Discussion	Total	Quality of the study
Chen et al. 2013	3	3	3	4	3	4	3	3	27	Medium
Saarela et al. 2013	4	4	4	4	3	3	4	4	30	Medium
El Hélou et al. 2014	4	3	3	4	3	4	4	4	29	Medium
Lauritano et al. 2019	4	3	3	2	3	3	2	3	23	Low
Craig & Johnsen 2020	4	4	4	3	4	4	4	4	31	Medium
Hakeem et al. 2021	4	4	4	3	3	4	4	4	30	Medium
Lopez-Jornet et al. 2021	4	4	4	4	3	4	3	3	29	Medium
Schmalz et al. 2021	3	3	3	4	4	4	4	3	29	Medium
Yang et al. 2021	4	4	3	4	4	4	3	4	30	Medium
Devika et.al. 2023	4	4	4	4	4	3	3	3	29	Medium
Mitsutake et al. 2023	4	4	3	3	3	3	3	4	27	Medium
Julkunen et al. 2024	4	4	3	4	3	4	4	4	30	Medium

### Synthesis of results

The qualitative thematic analysis of the selected articles resulted in the emergence of four main themes namely: risk factors for ROHD, attributes related to ROHD, challenges encountered in managing ROHD and management approaches for ROHD among older adults. The details of the main and sub-themes are provided in Table 4.

**Table 4.** Main themes and sub-themes emerging from qualitative analysis.

Main themes	Sub-themes
Risk factors for ROHD	Systemic factors ^16^^,^^17^^,^^19^^,^^20^^,^^22^^–^^24^^,^^26^^,^^27^
	Oral factors ^19^
	Socioeconomic factors ^20^^,^^21^^,^^27^
Attributes related to ROHD	Frailty ^16^^,^^17^^,^^21^^,^^27^
	Malnutrition ^17^^,^^18^^,^^21^^,^^23^^,^^25^^,^^27^
	Oral Frailty ^19^^,^^21^^,^^27^
	Oral health related factors ^16^^–^^20^^,^^22^^–^^27^
Challenges encountered in management of ROHD	Problems related to older adults ^17^^,^^19^
	Problems related to nursing staff ^16^^,^^17^^,^^19^
	Challenges in individualizing dental treatment plans ^16^
Management approaches for ROHD	Need for specific dental protocols in residential care institutions ^19^
	Oral health indicators to be included in routine geriatric assessments ^21^
	Routine dental visits, scaling and cleaning ^19^^,^^26^
	Dental care against periodontal disease ^24^^,^^26^
	Specific training for nursing staff for oral health maintenance ^17^^,^^19^^,^^24^
	Attention to nutrition in orally frail individuals ^23^
	Teaching Tool for dental students ^20^
	Training to caregivers to identify oral pain-related signs and behavioural changes ^16^^,^^22^

Risk factors for ROHD included systemic, socioeconomic factors and oral factors. Systemic factors included a wide array of systemic conditions mainly dementia, schizophrenia, and cardiovascular disorders among others. Oral factors included oral conditions such as xerostomia, hyposalivation, microbiota as well as level of oral self-care and oral frailty. Attributes related to ROHD comprised of frailty, malnutrition, oral frailty and oral conditions encountered. These attributes depicted a close linkage with one another as well as with the living conditions such as oral hygiene habits, institutionalization, edentulism, and oral health related quality of life (OHRQoL).

The next two themes were related to the challenges encountered in the management of ROHD and the potential solutions which could be implemented in the management of ROHD among older adults.

## Discussion

This scoping review intended to examine the available evidence on the rapid deterioration of oral health in the older population, with a special emphasis on identifying the research gaps. Additionally, the findings relating to the qualitative thematic analysis shed light on potentially promising approaches which could be imbibed into everyday interprofessional medical practices to better manage ROHD among older adults, thereby enhancing OHRQoL.

### Systemic conditions and oral health

There is a strong relationship between oral diseases and systemic diseases, with research demonstrating a robust connection between dental health and noncommunicable diseases such as cancer, neurodegenerative disorders, diabetes, heart disease, depression, inflammatory bowel disease, rheumatic diseases, obesity, stomach helicobacter pylori, and asthma. ^7^ It is important to note that the prevalence of several chronic medical disorders rises with age. ^8^

Dementia emerged as an important condition affecting the oral health condition among older adults in this scoping review. Poor oral hygiene in older people with dementia is caused by a number of variables, including behavioural issues, deteriorating cognitive function, degree of dementia and insufficient dental staff nursing education. ^19^^,^^22^ Similarly, neuropsychatric disorders like schizophrenia have a significant negative effect on people’s health. These patients have poor dental hygiene, which lowers their quality of life and raises their risk of systemic illnesses. ^24^

Systemic health and oral health share a bi-directional relationship with each other as can be observed in the available evidence, where oral health also tends to adversely affect the general health of individuals. The importance of regular dental check-ups in preventing acute hospitalization occurring due to diseases like pneumonia, cerebrovascular disease and urinary tract infections, has been highlighted by a retrospective study conducted in Japan on older adults included in this review. ^26^ The studies on diabetes and oral health has been always focused on the periodontal tissues and maintenance of glycemic control. ^28^ This is in line with evidence which suggests that early detection and treatment of oral health issues could avert poor oral health and a reduction in OHRQoL particularly in older adult patients with chronic illnesses. ^7^

### Nutrition and oral health

There is a reciprocal and dynamic relationship between dental health and nutrition. Poor dental health may increase an older adult person’s risk of malnourishment or malnutrition, particularly if they are institutionalized. ^21^^,^^23^ This further magnifies the importance of including oral health indicators in geriatric examinations. ^21^ Evidence suggests that a risk of nutritional deficiency was substantially correlated with a negative self-perception of oral status; however, this correlation vanished when socioeconomic level, neurosensory impairments, and chronic illnesses were taken into account. ^18^

### Frailty and oral health

Reduced stress tolerance, elevated susceptibility, and compromised functioning are all considered indicators of frailty. It raises the possibility of hospitalization, disability, falls, and death. Oral Frailty (OFr), which is defined in the literature as “weakness and fatigue of the oral muscles,” contains elements including poor oral health, impaired muscle performance, the possibility of weight loss, issues with chewing and swallowing, changes in the food’s composition, trouble speaking, and tongue or tooth pain. One of the included studies suggested that protection against OFr may come from maintaining one’s natural teeth, maintaining good oral health throughout one’s life, and preventing oral illnesses. ^27^

### Management approaches for ROHD in older adults

Institutionalized older adults need assistance in doing oral hygiene activities ^17^ and usage of various oral hygiene aids such as adaptive toothbrush handles, collis-curve brushes, triple-headed toothbrushes, water flossers, interdental brushes of different diameters, floss holders/single-use floss picks, and other oral hygiene products like electric toothbrushes help these people remove plaque more effectively and help with physical adaptations. ^6^^,^^9^

Among other potential approaches to manage ROHD in older adults, the need for specific dental protocols to be followed in residential care institutions along with training for nursing staff to cater to oral health maintenance and empowering caregivers in identifying oral pain-related signs were highlighted in the included articles. In particular, attention to nutritional needs in orally frail individuals as well as providing adequate dental care against periodontal disease emerged as important approaches. Another study discussed a simple yet effective teaching tool for dental students to provide care to the geriatric population and people with special needs. ^20^

### Future directions

The findings of the present review warrant more high quality research in this important area with rising geriatric population worldwide. This research also lays down potentially promising approaches, which can be researched as well as implemented with regards to the management of ROHD among aging adults.

## Conclusion

Age-related dental disease has a negative effect on overall health and quality of life. In order to avoid dental disorders and to maintain and improve oral health in older adults, an integrated and multidisciplinary approach is essential. If ROHD is not treated, it may lead to poor health, a lower quality of life, and in severe cases, systemic infections that increase hospitalizations and possibly cause death.

## Data Availability

All data underlying the results are available as part of the article and no additional source data are required. Figshare: PRISMA ScR guidelines checklist Shenoy, R. Understanding Rapid Oral Health Deterioration and its associated factors among Older Adults-A Scoping Review. figshare. Dataset. 2024 https://doi.org/10.6084/m9.figshare.25360291.v5. ^29^
