# Supplementary material for: Understanding rapid oral health deterioration and its associated factors among older adults: A scoping review
Source: F1000Res. 2024 Apr 17;13:284. [Version 1] doi: 10.12688/f1000research.149120.1 (PMC11143401; doi:10.12688/f1000research.149120.1)
Supplement: Peer review history

# Reviewer report: Reviewer response for version 1

**DOI:** 10.5256/f1000research.163534.r272000

**Published:** 2024-05-31

**Authors:** Rohit Nair

**Affiliations:** 1 Department of Preventive and Community Dentistry, The University of Iowa College of Dentistry and Dental Clinics, Iowa City, IA, USA

As a clinical concept, Rapid Oral Health Deterioration (ROHD) is tangible but difficult to define. What constitutes "deterioration" and the time frame for such change can vary from person to person but it can be agreed that oral disease negatively impacts the frail older adult's quality of life. It is imperative to address dental caries, periodontal disease as needs arise to avoid undesired sequelae. In this article, Ranjith and colleagues provide a detailed  review of emerging thought about oral health deterioration seen in medically frail older adults. Their approach seems to be methodologically robust and reproducible, and care has been taken at every step to ensure inclusion of the best available literature that pertains to their specific topic of interest. Table 4 neatly summarizes the key themes that one might encounter when dealing with patients who are either at risk for, are experiencing or have experienced ROHD.

 Although we may accept a general association between oral and systemic disease as proposed here, the issue is a rather complex one. As suggested by Philstrom et al, it is sufficient to promote good oral health as a benefit in and of itself  rather than because it carries a specific systemic health benefit [Ref-1]. The current article might present a more balanced view if it referenced controversies in this area and the limitations inherent in studying associations between oral and systemic health. 

 Overall, a very good report .

Are the rationale for, and objectives of, the Systematic Review clearly stated?

Yes

Is the statistical analysis and its interpretation appropriate?

I cannot comment. A qualified statistician is required.

If this is a Living Systematic Review, is the ‘living’ method appropriate and is the search schedule clearly defined and justified? (‘Living Systematic Review’ or a variation of this term should be included in the title.)

Not applicable

Are sufficient details of the methods and analysis provided to allow replication by others?

Yes

Are the conclusions drawn adequately supported by the results presented in the review?

Yes

Reviewer Expertise:

Geriatric Dentistry, Dentistry for Adults with Special Health Care Needs

I confirm that I have read this submission and believe that I have an appropriate level of expertise to confirm that it is of an acceptable scientific standard.

## References

[1] : Promoting oral health care because of its possible effect on systemic disease is premature and may be misleading. *The Journal of the American Dental Association* .2018;149(6) : 10.1016/j.adaj.2018.03.030 401-403 10.1016/j.adaj.2018.03.030 29804570

# Reviewer report: Reviewer response for version 1

**DOI:** 10.5256/f1000research.163534.r271999

**Published:** 2024-05-28

**Authors:** Tarakant Bhagat, Naresh Prasad Joshi

**Affiliations:** 1 Department of Public Health Dentistry, College of Dental Surgery, B.P. Koirala Institute of Health Sciences, Dharan, Nepal; 2 Community Dentistry, Chitwan Medical College, Chitwan, Bagmati, Nepal

The study explores a compelling case of sharp decline in oral health in geriatric populations because of the complex interplay between systemic and oral health.  Congratulations to the authors for their intriguing work!

 We feel there are few facets in this research that can be framed in a finer way.

1. One of the objective is to identify knowledge gaps in research. This could have been discussed further in the report.
2. Under information sources and search strategy in the Methods section, it appears as if keywords have been repeated. ( ‘older adults’, ‘older adults’)
3. Specifying the limitations of the scoping review process may be considered.

Are the rationale for, and objectives of, the Systematic Review clearly stated?

Yes

Is the statistical analysis and its interpretation appropriate?

Yes

If this is a Living Systematic Review, is the ‘living’ method appropriate and is the search schedule clearly defined and justified? (‘Living Systematic Review’ or a variation of this term should be included in the title.)

Yes

Are sufficient details of the methods and analysis provided to allow replication by others?

Yes

Are the conclusions drawn adequately supported by the results presented in the review?

Yes

Reviewer Expertise:

Dentistry, oral health, geriatric health, dental caries, oral cancer

We confirm that we have read this submission and believe that we have an appropriate level of expertise to confirm that it is of an acceptable scientific standard.
